# Progress in Dermatology

**Published:** 1901-10-19

**Authors:** 


### PROGRESS IN DERMATOLOGY.

**Bacteriology.**—A most interesting discussion was introduced by Sabouraud[1] at Cheltenham. He laid down that the most important forms of cocci are (*a*) the streptococcus of Fehleisen, which produces itself contagious impetigo ; (*b*) the yellow staphylococcus which causes the impetigo of Bockhart ; and (*c*) the grey staphylococcus (Unna's morococcus) causing simple pityriasis or seborrhœic eczema. Each of these forms may also, under special conditions or with the help of other organisms, produce other disorders. Thus we may find the first in pemphigus, erythema nodosum, erysipelas, eczema, Bateman's rupia ; and the best remedy for any superficial lesion produced by it is a 1-per-cent. solution of zinc sulphate. The yellow staphylococcus leads to boils, sycosis, acne picis, and may complicate eczema. The best remedy is a sulphur lotion of, say, 19 per cent. Against the lesions due to the morococcus, oil of cade with the yellow oxide of mercury is the best local remedy. Galloway, while accepting the streptococcus as the cause of erysipelas, elephantiasis, and chronic œdema, thinks that the white staphylococcus is joined with it in producing the impetigo of children. The staphylococci vary enormously as to virulence, though probably forming only one species ; even the morococcus under cultivation may be changed into S. albus.

**Seborrhœa.**—Colcott Fox[2] reviews the history of scientific teachings on this subject and the difficulties of distinguishing the functions of the sebaceous and sweat glands. Unna held that the disease is due to a specific inflammation produced by micro-organisms which stimulate the sweat glands to produce oil, while Sabouraud gives reasons for ascribing oily seborrhœa and baldness to an infection of sebaceous glands. Polymorphic acne is a complication of this oily seborrhœa. Sabouraud[3] moreover separates entirely all forms of pityriasis from seborrhœa, as the latter is always and exclusively fatty and due to a bacillus. This is found pure in the fatty plugs which can be expressed from a seborrhœic skin. Some few of these plugs are transformed into comedones, and these latter by a secondary infection with various cocci produce different types of acne. Bacillary seborrhœa of the scalp is the common cause of baldness. On the other hand pityriasis, though often occurring with seborrhœa, is due to a coccus and the flask bacillus and gives rise to superficial scales. Thus Unna's seborrhœic eczema is a combination of two distinct diseases. Gottheil[4] gives an account of a case of pityriasis versicolor occurring on the face only. The patient was a negro and the spots looked at first like leucoderma, and were slightly scaly and greyish-white in colour. He also refers to a case where the affection appeared on the palms. In either situation he thinks it is extremely rare. Though J. Sobel holds that the lesions are not infrequently seen on the face[5] and mistaken for syphilis, he points out that[6] Allen's iodine-test will show up pale and almost invisible lesions, and distinguish the disease from other non-parasitic affections. Hyperidrosis, rather than the general ill-health of the patient, is the most important predisposing cause : hence it frequently occurs in phthisis when night sweats are excessive, but it is by no means unknown in persons of perfect health.

**Lichen.**—M. Dockrell[7] argues that pityriasis rubra pilaris is entirely distinct from lichen, and that the latter passes through four stages—the acuminate, the planus, the hypertrophic, and the verrucose forms, which are often held to be distinct diseases. While lichen in the earlier stages does not, as he thinks, affect the hair follicles or the sweat glands, in P. pilaris the hair follicles are primarily involved and there is no tendency to the formation of hyaline material. This hyaline degeneration is an important element in lichen, but the differences in colour and shape of the papules, which are often taken as the basis of distinct varieties, are unimportant except as indicating the stage of the disease. Lichen spinulosus is the name given by J. Hutchinson[8] to a form where little spines of hardened sebaceous matter arise from the hair follicles. He regards it as a tuberculous affection allied to the various other types of L. scrophulosus. It must be distinguished from ichthyosis, and rarely occurs in the subjects of phthisis, though the sufferers have generally a strongly marked family history of tubercle.

**Eczema.**—Malcolm Morris[9] notices the frequency with which eczema commences in children around the seborrhœal patch so often seen on the vertex at birth. Care should be taken not to irritate this spot by soap or other means of removing the crust. When the dry eczema has spread to the body a very mild application, such as 5 grains of sulphur in one ounce of ointment, is all that is required. Among the agents which convert a dry seborrhœal rash into an acute moist eczema are vaccination, measles and intestinal worms, but the pathology is quite unknown. At this stage he recommends a dose of calomel, a dusting powder, then carron oil with zinc and lanoline, and later on a weak ammoniated mercury ointment. In adults an acute sudden attack is best treated with calomel, tartrated antimony, and dusting powder ; intertrigo with an antiseptic lotion and a weak sulphur ointment ; varicose eczema with Unna's zinc glycerine jelly, or in very chronic cases salicylic resorcin or pyrogallic ointment. The eczema which occurs at the menopause, chiefly on the head and face, is best treated by ichthyol given internally, in doses gradually increased from two to ten grains three times a day, and with strong ointments of sulphur or resorcin. Another form seen at this period is the very acute eczema of the vulva and anus. Severe eczema in very advanced life demands the free use of opium. H. Waldo[10] insists on the need of scrupulous cleanliness in treatment. Every day the parts should be washed with soft water to which oatmeal and bicarbonate of soda or a mild antiseptic may be added. Immediate drying with a soft towel is essential. The healthy parts of the body should be washed daily with soap and water on account of the sympathetic action of one part of the cutaneous area over the rest. In the choice of local remedies the main thing is to protect the part from the air and other irritants. Though we do not find marked benefit from strict dieting, yet some attention should be paid to the general health as influencing the soil on which the germs flourish. Thus aperients, blue pill, and sometimes the addition of milk to the meals, and abstention from alcohol, are of value.

[1] Brit. Med. Jour., Sept. 21. [2] Brit. Med. Jour., Sept. 28. [3] Ibid. [4] Med. Rec., April 27. [5] Ibid. [6] Med. Rec., June 8. [7] Brit. Med. Jour. [8] Clin. Jour., June 12. [9] Lancet, May 4. [10] Brit. Med. Jour., Sept. 21.

